# How the Oocyte Nucleolus Is Turned into a Karyosphere: The Role of Heterochromatin and Structural Proteins

**DOI:** 10.3390/jdb12040028

**Published:** 2024-10-18

**Authors:** Venera Nikolova, Maya Markova, Ralitsa Zhivkova, Irina Chakarova, Valentina Hadzhinesheva, Stefka Delimitreva

**Affiliations:** Medical Faculty, Department of Biology, Medical University of Sofia, 1431 Sofia, Bulgaria; m.markova@medfac.mu-sofia.bg (M.M.); r.zhivkova@medfac.mu-sofia.bg (R.Z.); i.chakarova@medfac.mu-sofia.bg (I.C.); v.hadzhinesheva@medfac.mu-sofia.bg (V.H.); s.delimitreva@medfac.mu-sofia.bg (S.D.)

**Keywords:** cytoskeleton, germinal vesicle, meiosis, meiotic spindle, karyosphere, nuclear envelope, nuclear lamina, oocytes

## Abstract

Oocyte meiotic maturation includes large-scale chromatin remodeling as well as cytoskeleton and nuclear envelope rearrangements. This review addresses the dynamics of key cytoskeletal proteins (tubulin, actin, vimentin, and cytokeratins) and nuclear envelope proteins (lamin A/C, lamin B, and the nucleoporin Nup160) in parallel with chromatin reorganization in maturing mouse oocytes. A major feature of this reorganization is the concentration of heterochromatin into a spherical perinucleolar rim called surrounded nucleolus or karyosphere. In early germinal vesicle (GV) oocytes with non-surrounded nucleolus (without karyosphere), lamins and Nup160 are at the nuclear envelope while cytoplasmic cytoskeletal proteins are outside the nucleus. At the beginning of karyosphere formation, lamins and Nup160 follow the heterochromatin relocation assembling a new spherical structure in the GV. In late GV oocytes with surrounded nucleolus (fully formed karyosphere), the nuclear envelope gradually loses its integrity and cytoplasmic cytoskeletal proteins enter the nucleus. At germinal vesicle breakdown, lamin B occupies the karyosphere interior while all the other proteins stay at the karyosphere border or connect to chromatin. In metaphase oocytes, lamin A/C surrounds the spindle, Nup160 localizes to its poles, actin and lamin B are attached to the spindle fibers, and cytoplasmic intermediate filaments associate with both the spindle fibers and the metaphase chromosomes.

## 1. Peculiarities of Mammalian Prophase I Oocytes

At the end of meiotic prophase I, mammalian oocytes, similarly to the oocytes of many other animals, undergo a long arrest. When hormonal signals trigger meiotic resumption, prophase–metaphase transition and the arrangement of the meiotic spindle begin. The process requires large-scale chromatin remodeling and profound changes in nuclear architecture. The behavior of cytoskeletal elements during these steps of oocyte meiosis is very specific. This specificity is due to the following facts [1,2,3,4]:

- Oocytes are very large cells, even in mammals. For example, the mouse oocyte is 70–80 µm in diameter and the human oocyte is 100–120 µm.
- The nucleus occupies an extremely large territory: its absolute diameter is about 25–30 µm in the mouse and up to 40 µm in the human.
- The centrosomes have been destroyed during prophase I, so the formation of microtubules depends on acentriolar microtubule organizing centers (MTOCs). Chromosomes themselves direct the formation of multiple active MTOCs that later assemble the meiotic spindle.
- Because the distance of action of microtubules is limited (they can effectively bind to chromosomes only in cells with a diameter not exceeding 30 µm), the positioning of chromosomes is helped by other types of cytoskeletal elements, namely, microfilaments and intermediate filaments.
- Long before nuclear envelope breakdown (about 4 h), nuclear pores lose their function to be checkpoints for nucleocytoplasmic transport. Large openings in the envelope (up to 800 nm in diameter, reflecting the large size of the nucleus itself) appear and provide access for cytoplasmic cytoskeletal fibers to the chromatin [3].
- At that period, the shape of the nucleus remains visibly unchanged, but the free penetration of cytoplasmic components means that it has effectively stopped being a separate compartment of the cell.

The nucleus of the immature prophase I oocyte is traditionally called a germinal vesicle (GV). This term was introduced by Purkinje in 1825 for the nucleus of the hen egg, but he considered it a cell that develops into an embryo [5]. Later, in 1834, Bernhardt (Purkinje’s doctoral student) used the term GV to describe the nucleus in mammalian oocytes. The tradition to use “GV” instead of “nucleus” is still alive, maybe because embryologists always intuitively knew that the GV is not an ordinary cell nucleus. Now, knowing the unique behavior of the oocyte GV, it seems that the decision to keep the old name in use has been appropriate.

## 2. The “Surrounded Nucleolus” (Karyosphere) of GV Stage Oocytes

Inside the unusual nucleus of the GV oocyte resides an even more unusual nucleolus. It was mentioned for the first time in 1835 as *macula germinativa* (“germinal spot”), an absolutely round and transparent structure in the GV [5,6]. Since then, this structure has also been called “nucleolus-like body” [7], “atypical nucleolus” [8], or just “nucleolus”, although it actually stops functioning as a nucleolus [9]. The consecutive changes in its appearance during the meiotic prophase I are related to changes in chromatin arrangement: step by step, heterochromatin aggregates around the nucleolus, eventually encircling it with a perfect sphere. In the literature, in the initial stage when the nucleolus is fairly typical and transcriptionally active, it is known as a “non-surrounded nucleolus” (NSN), and in the later stage when it loses its transcriptional activity and acquires its heterochromatin sphere, as a “surrounded nucleolus” (SN), e.g., [10]; “rimmed nucleolus” [11]; or “karyosphere” [12]. Some authors also use the terms “partially nonsurrounded nucleolus” (pNSN) and “partially surrounded nucleolus” (pSN) for the intermediate stages [13]. In the present review, the term “karyosphere” is preferred because of its brevity and implication that this structure no longer performs the standard nucleolus functions.

Shortly after the SN stage, the oocyte nucleus loses its borders and disassembles. This process is called germinal vesicle breakdown (GVBD) and marks the transitional period between prophase I and metaphase I. At some point during the GVBD stage, the karyosphere is disassembled [13]. To summarize, the events characterizing late GV and GVBD stages are gradual chromatin condensation, karyosphere formation, nuclear envelope permeabilization and breakdown, and karyosphere breakdown. This is followed by meiotic spindle assembly, migration, and anchoring to the oolemma (metaphase I); the extrusion of the first polar body; and metaphase II.

Karyosphere formation has been found to be an indicator of oocyte meiotic and developmental competence: only oocytes with karyosphere status corresponding to their cytoplasmic maturation stage can resume meiosis, assemble a proper spindle, mature to metaphase II and, if fertilized, develop beyond the first mitotic division of the zygote. This has been found to be valid for the oocytes of different mammalian species such as humans [8], mice [14,15], and cattle [16]. In this respect, it may be significant that NSN-to-SN transition, i.e., karyosphere formation, is accompanied by a change in the specific pattern of epigenetic markers (modified histones) in mouse [17], porcine [18], and human oocytes [19].

The main function of the karyosphere is to join oocyte chromosomes together and thereby ensure that no bivalent will be lost by the meiotic spindle [20]. In the late GV stage, the oocyte chromatin changes its state from a transcription template responsible for the maintenance and growth of a giant cell to condensed chromosomes that can be arranged and segregated accurately and occupy only a very limited portion of the nuclear volume. Although chromatin condensation at this stage proceeds along the entire chromosome length, the most important restructuring processes take place in and around the karyosphere: all of the centromeres in mouse SN oocytes are associated with the karyosphere, whereas in NSN oocytes, only the chromosomes possessing nucleolus-organizing regions have a perinucleolar position [21].

The karyosphere is an evolutionarily conserved structure observed not only in mammals but also in various other vertebrates and some invertebrates [20]. Nevertheless, in different animals it may be organized differently; e.g., in rabbit SN oocytes, the condensed chromatin is at first netlike, then forms several small clumps, and finally coalesces into a single large clump. Among primates, while the oocytes of the studied monkeys form a rim of condensed chromatin at the nucleolar surface as in mice, human oocytes are more similar to those of rabbits. In goats, condensed chromatin remains in the configuration of clumps, while in cattle, pigs, and horses, it surrounds the nucleolus but usually forms an incomplete (e.g., horseshoe-shaped) rather than a complete sphere [13,22].

In addition, there is a group-specific variation in the relative positions of the chromosomes and the extrachromosomal structural elements. Some groups, such as certain insects and amphibians, have a karyosphere surrounded by a capsule, while others, notably humans and other mammals, have an “inverted karyosphere” in which the condensed chromosomes are located on the outside with respect to a central body. It is hypothesized that the extrachromosomal elements (capsule or central body) may play a wider role in the compartmentalization of nuclear functions than just a mechanical scaffold for chromosomes [20]. However, in all cases, the karyosphere formation is accompanied by a dramatic collapse of the nuclear region occupied by the chromatin.

At the same time, the nucleolus loses its function as a site of ribosomal RNA synthesis. The chromosomes gradually encircle its inactivated remnant. They contribute to the building of a new structure at the same place by surrounding its edge with their heterochromatin centromeric regions [5,22]. This way, while in early GV oocytes the heterochromatin is predominantly associated with the nuclear periphery, as in somatic cells [23], in late GV oocytes it is organized by the karyosphere. This front-line shifting assembles the chromosomes together in a smaller volume and prepares them for their congression and then migration to the oocyte periphery. A question arises whether the relocation of heterochromatin correlates with a concomitant relocation of the proteins involved in nuclear envelope maintenance and/or meiotic spindle assembly.

The subject of this short review is the dynamics of several key proteins related to large-scale chromatin remodeling in mouse oocytes during the processes mentioned above, with special attention to karyosphere formation. Chronologically, these processes belong to the oocyte meiotic maturation, i.e., the late stages of meiosis—from meiotic resumption after the long arrest in prophase I to full maturation and a second arrest at metaphase II. The nuclear proteins that will be discussed are lamins B and A/C, as well as the nuclear pore complex component nucleoporin 160 (Nup160). The cytoplasmic cytoskeletal proteins tubulin and actin, and the cytoplasmic intermediate filament (IF) components cytokeratins and vimentin, also undergo stage-specific reorganizations and can be used to detect permeability changes in the nuclear envelope before its visible breakdown at the GVBD stage. Our team has studied the behavior of these proteins in differentiating mouse oocytes for years, and photos of our work will be used to illustrate the text.

## 3. Early GV Oocytes Without Karyosphere (Non-Surrounded Nucleolus)

**Figure 1:**
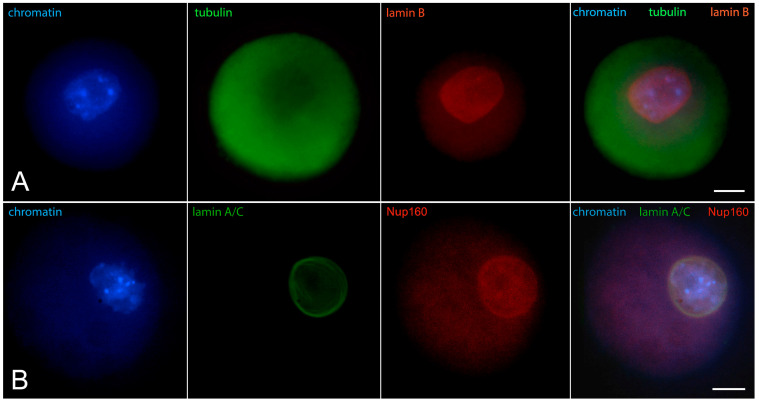
Early GV mouse oocytes before the formation of karyosphere: (**A**) reaction for lamin B, alpha-tubulin, and chromatin; (**B**) reaction for lamin A/C, Nup160, and chromatin, epifluorescence. These cells were labeled as a part of the research described in [24]. Chromatin is stained with Hoechst 33258 and the proteins are visualized by indirect immunofluorescence. Bar = 20 μm.

**Figure 2:**
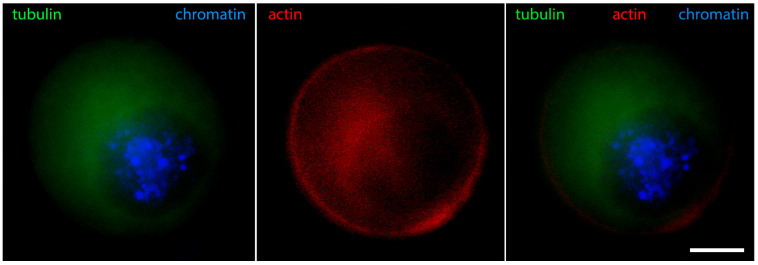
Mouse oocyte in the GV stage before karyosphere formation—reaction for alpha-tubulin, fibrillar actin, and chromatin, epifluorescence. The cell was labeled as a part of the research described in [29]. Actin is visualized using labeled phalloidin. Bar = 20 μm.

Early GV oocytes without a visible karyosphere are prophase-arrested cells that have not yet resumed meiosis [14]. In them, the configuration of chromatin and the distribution of nuclear and cytoplasmic proteins largely follow the pattern described for typical somatic cells. Heterochromatin is observed predominantly at the nuclear periphery. Lamins and Nup160 are restricted to the nuclear envelope [24], as shown in Figure 1. Of these nuclear envelope proteins, the best studied in oocytes is lamin A/C. In mice, it has recently been shown to dissociate rapidly from the nuclear envelope in oocytes from aged females [25], and to include the germ cell-specific isoform lamin C2 found also in spermatocytes [26]. Lamin A/C has also been localized in the oocytes of other mammals, such as cattle [27]. Tubulin, due to the absence of centrosomes and the initially inactive state of acentriolar MTOCs, is localized diffusely in the ooplasm but shows a tendency to concentrate around the germinal vesicle [28]. Actin is also found throughout the cytoplasm but preferentially localized to the cortex. At this stage, the distribution of cytoplasmic intermediate filaments (cytokeratin and vimentin) overlaps with that of fibrillar actin (Figure 2) [29].

## 4. GV Oocytes in the Process of Karyosphere Formation (Partially Non-Surrounded and Partially Surrounded Nucleolus)

**Figure 3:**
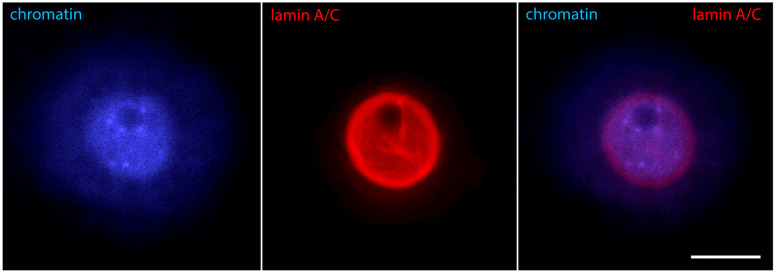
Mouse oocyte in the GV stage during karyosphere formation—reaction for lamin A/C and chromatin, epifluorescence. The cell was labeled as a part of the research described in [24]. Bar = 20 μm.

As oocyte maturation proceeds, chromatin is initially still dispersed in the whole GV volume, but regions of condensed chromatin appear around the nucleolus in parallel with the gradual cessation of its standard functions of rRNA synthesis and ribosome biogenesis [30]. This is the beginning of karyosphere formation. Of the proteins discussed here, the first one that changes its distribution is lamin A/C. At that period, it is detected not only in the nuclear periphery but also inside the nucleus, and only the inner volume of the karyosphere remains free of lamin A/C [24] (Figure 3).

**Figure 4:**
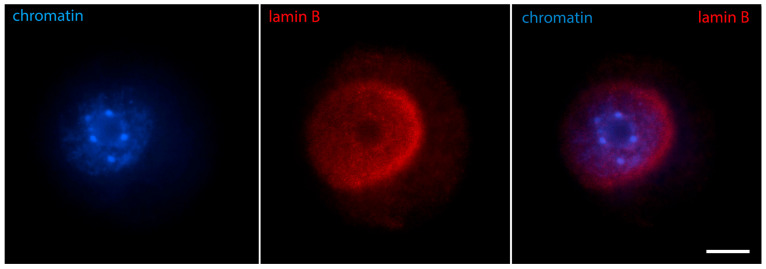
Mouse oocyte in the GV stage during karyosphere formation—reaction for lamin B and chromatin, epifluorescence. The cell was labeled as a part of the research described in [24]. Bar = 20 μm.

**Figure 5:**
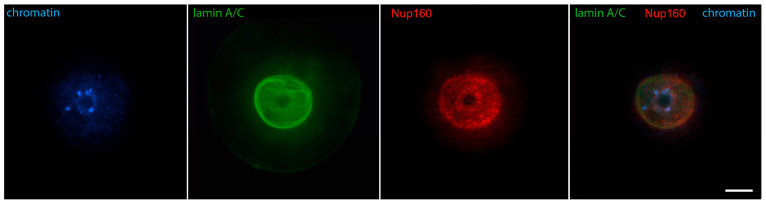
Mouse oocyte in the GV stage during karyosphere formation—reaction for lamin A/C, Nup160, and chromatin, epifluorescence. The cell was labeled as a part of the research described in [24]. Bar = 20 μm.

At a slightly later stage of karyosphere formation, when almost all the heterochromatin blocks are arranged in a sphere, lamin A/C is joined by lamin B (Figure 4) and Nup160 (Figure 5). At that stage, immunofluorescent reaction for both lamins and Nup160 is still positive at the GV periphery, as expected, and also begins to appear at the heterochromatin rim. Microscopically, this situation is observed as two spheres, the outer one corresponding to the nuclear envelope and the inner one to the surface of the karyosphere. A diffuse reaction for lamins and Nup160 can be registered between the two spheres. Although the nuclear envelope has not yet disassembled, some of its elements are gradually transferred to the inner nuclear compartment. Hence, during karyosphere formation, lamins and Nup160 follow the relocation of heterochromatin and assemble a new spherical structure inside the GV [24,31].

The immunolocalization of lamins B and A/C and the nuclear pore complex component Nup160 first at the nuclear periphery, and then inside the nucleus, and particularly at the karyosphere, shows that these nuclear envelope components, even at the early stages of karyosphere formation, are no longer restricted solely to the nuclear periphery. This suggests subtle changes in the structure and, presumably, the function of the nuclear envelope, coinciding with the resumption of meiosis manifested by the condensation of perinucleolar chromatin. The partial relocation of lamins and nucleoporin 160 to the nuclear interior indicates that the nuclear envelope is gradually depleted of the protein components responsible for its mechanical strength and normal permeability. The slightly later shifting of lamin B compared to lamin A/C could be explained by its more intimate association with the inner nuclear membrane [32].

The reorganization of chromatin and the nuclear envelope is accompanied by the reorganization of tubulin in the cytoplasm. In mice, numerous MTOCs containing γ-tubulin and pericentrin associate with the outer surface of the nucleus and become active in microtubule nucleation [11,33]. This process may show species-specific differences; recently, a structure provisionally named “human oocyte microtubule organizing center” has been observed in human oocytes. It first appears beneath the cell cortex, migrates to the nuclear envelope, and fragments after GVBD to associate with the kinetochores and participate in spindle assembly [34].

## 5. GV Oocytes with Fully Formed Karyosphere (Surrounded Nucleolus)

The large-scale chromatin reorganizations associated with karyosphere formation must be coordinated with changes in the nuclear envelope. Chromosomes detach from it as it becomes unstable and finally disintegrates, marking the GVBD stage and the transition to metaphase I. During this process, the nuclear lamina serving as the mechanical support of the nuclear envelope collapses. Even before the apparent breakdown of the nuclear envelope as a microscopic structure, its permeability increases as large openings appear within it, with a diameter exceeding that of nuclear pores by 10–20 times, i.e., by an order of magnitude [3]. Similar changes have been described for the nuclear envelope of somatic cells entering mitosis [35].

As a result of the gradual loss of nuclear envelope integrity, the redistribution of nuclear envelope proteins in GV oocytes with an emerging karyosphere is followed by a more striking redistribution of the cytoplasmic proteins in oocytes with a well-formed karyosphere. At this stage, cytoplasmic proteins such as the cytoskeletal components can be detected inside the nucleus [24]. Although still preserved as a microscopic structure, the nuclear envelope apparently stops functioning as a barrier for macromolecules. The observed mixing of cytoplasmic and nuclear proteins reveals that the late germinal vesicle becomes a “phantom nucleus”. It should be remembered that this change which prepares the nuclear envelope for its imminent breakdown comes only after the significant relocation of Nup160. The fact that the detachment of an important component of the nuclear pore complexes coincides with an increase, rather than a decrease, of nuclear envelope permeability implies that this permeability is no longer dependent on nuclear pores, and is instead mediated by some broader openings, presumably those described in [3,35]. Data from the mitotic prophase of somatic cells indicate that the phosphorylation of nucleoporins under the control of Cdk1–cyclin B leads to an increase in nuclear envelope permeability as a result of partial nuclear pore complex disassembly, and that the envelope is further compromised by microtubule-mediated tearing [36].

**Figure 6:**
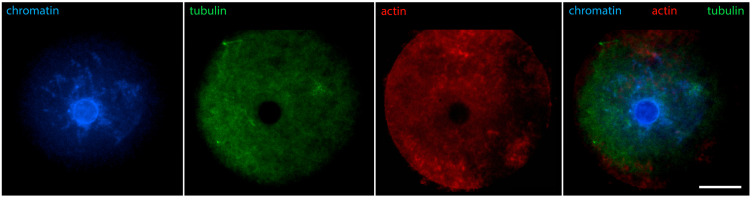
Mouse oocyte in the GV stage with well-contoured karyosphere—reaction for alpha-tubulin, fibrillar actin, and chromatin, epifluorescence. The cell was labeled as a part of the research described in [29]. Bar = 20 μm.

**Figure 7:**
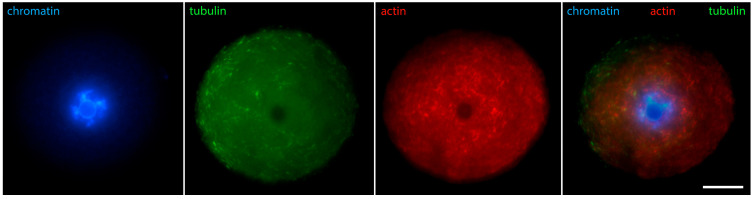
Mouse oocyte at the GV stage with fully formed karyosphere and condensed chromosomes—reaction for alpha-tubulin, fibrillar actin, and chromatin, epifluorescence. The cell was labeled as a part of the research described in [29]. Bar = 20 μm.

Figure 6 shows that in oocytes with chromosomes still relaxed, but the contour of the karyosphere already clearly visible as a ring of heterochromatin, the distribution of tubulin and fibrillar actin changes and the nucleus becomes positive for them. Only the interior of the karyosphere does not react. The most intensive reaction for actin and tubulin colocalizes with the most condensed chromatin. This pattern of actin and tubulin distribution is preserved in advanced GV oocytes with condensed chromosomes detached from the nuclear envelope (Figure 7). In this respect, it is noteworthy that in human and porcine oocytes at a slightly later stage, just before meiotic spindle assembly, actin-driven chromosome clustering has been reported [37].

**Figure 8:**
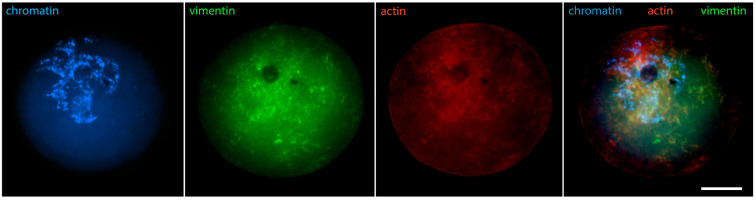
Mouse oocyte in the GV stage with fully formed karyosphere and condensed chromosomes—reaction for chromatin, vimentin, and fibrillar actin, epifluorescence. The cell was labeled as a part of the research described in [29]. Bar = 20 μm.

Cytoplasmic IF proteins demonstrate similar dynamics: during karyosphere assembly, they enter the nucleus and associate closely with heterochromatin. As a result, the brightest reaction for IFs is detected at the karyosphere surface [29]. Immunostaining for cytokeratins and vimentin at this stage reveals identical patterns (Figure 8 shows vimentin). These changes in the localization of cytoplasmic proteins indicate a dramatic increase in nuclear envelope permeability, allowing macromolecules without a nuclear localization signal to enter the germinal vesicle.

Hence, the observations of GV oocytes with a well-formed karyosphere show that the only region in the cell where cytoplasmic proteins are not found at this stage is the karyosphere interior. This is a result of karyosphere heterochromatin serving as an effective barrier to the penetration of proteins, at least in mouse oocytes [7].

**Figure 9:**
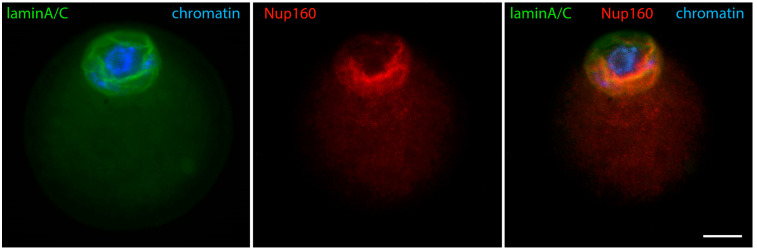
Mouse oocyte in the late GV stage with visible signs of nuclear envelope disintegration—reaction for lamin A/C, Nup160, and chromatin, epifluorescence. The cell was labeled as a part of the research described in [24]. Bar = 20 μm.

The increased permeability of the nuclear envelope allowing the access of cytoplasmic proteins to the GV interior is soon followed by its gradual disintegration. This change is illustrated by the deformations of the surface occupied by lamins and Nup160 (Figure 9) [24]. The gamma-tubulin-containing MTOCs have been reported to associate with these surface irregularities of the nuclear lamina, especially in the region overlying the karyosphere. When chromosomes are fully condensed, MTOCs relocate from the external to the internal surface of the collapsing lamina where the microtubules nucleated by them can interact with the chromosomes [33].

## 6. GVBD and Prometaphase I Oocytes

**Figure 10:**
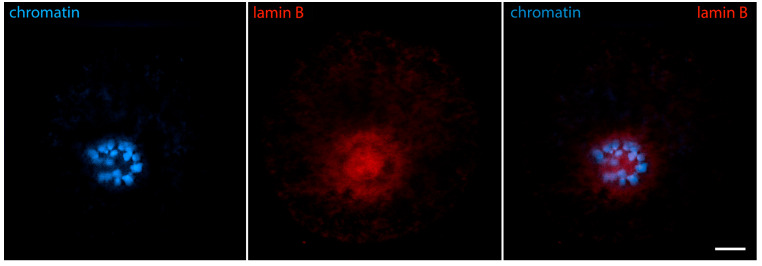
Mouse oocyte in the GVBD stage—reaction for lamin B and chromatin, confocal microscopy. The chromosomes are arranged in a prometaphase belt. The cell was labeled as a part of the research described in [24]. Bar = 10 μm.

The beginning of prometaphase I, traditionally named the germinal vesicle breakdown (GVBD) stage, is characterized by advanced chromatin condensation and the complete disintegration of the nuclear envelope. The karyosphere also gradually disintegrates, though it remains structurally recognizable for some time in the mouse [11] and other mammals [13]. Condensing chromosomes briefly form a characteristic configuration known as the prometaphase belt [38]. As a result of the karyosphere disassembly, its interior becomes accessible. At this stage, it shows a positive reaction for lamin B, pointing to a possible association of lamin B with centromeric heterochromatin [31] (Figure 10). However, cytoplasmic IF proteins, lamin A/C, Nup160, tubulin, and actin still do not enter inside the karyosphere. Instead, they stay attached to its periphery and the chromosomes [24].

The above-described changes in the localization of the cytoskeletal and nuclear envelope proteins correspond well to the changes in the organization and appearance of the karyosphere. This raised the question of how the dynamics of perinucleolar heterochromatin inside the nucleus is related to the gradual changes at its periphery. It is possible that these two processes are independently regulated during the GV progression. However, a more direct connection could also be hypothesized, with the gradual destabilization of the nuclear envelope due to the detachment of chromosomes from it to take part in karyosphere assembly.

**Figure 11:**
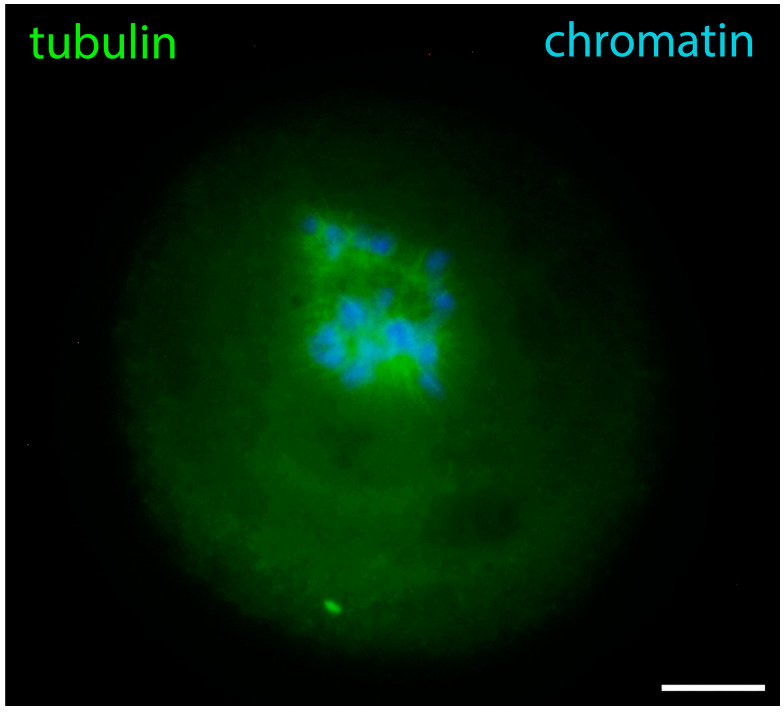
Prometaphase I mouse oocyte showing chromosomes and alpha-tubulin in the process of the construction of meiotic spindle, epifluorescence. The cell was labeled as a part of the research described in [29]. Bar = 20 μm.

GVBD is a transitional phase that prepares the meiotic spindle assembly. The extremely large volume of ooplasm and lack of centrioles make the process very specific for oocytes. Initially, multiple tubulin asters attached to the chromosomes appear [11]. This moment is captured in Figure 11. Later, the active MTOCs coalesce into two gamma-tubulin-containing foci forming the spindle poles [33]. During the arrangement of the meiotic spindle, lamin A/C surrounds the spindle, Nup160 surrounds its poles, actin and lamin B stay attached to spindle fibers, and cytoplasmic IFs contact both spindle fibers and metaphase chromosomes. In the oocytes of other mammals, there may be differences in tubulin reorganization, as evidenced by the recent discovery of the above-mentioned human oocyte microtubule organizing center [34].

## 7. Metaphase Oocytes

**Figure 12:**
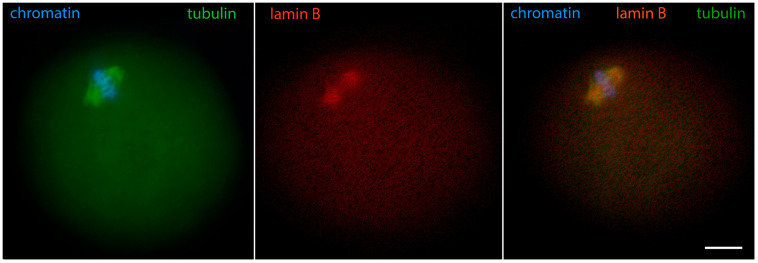
Metaphase I mouse oocyte stained for lamin B, alpha-tubulin, and chromatin, epifluorescence. The cell was labeled as a part of the research described in [24]. Bar = 20 μm.

**Figure 13:**
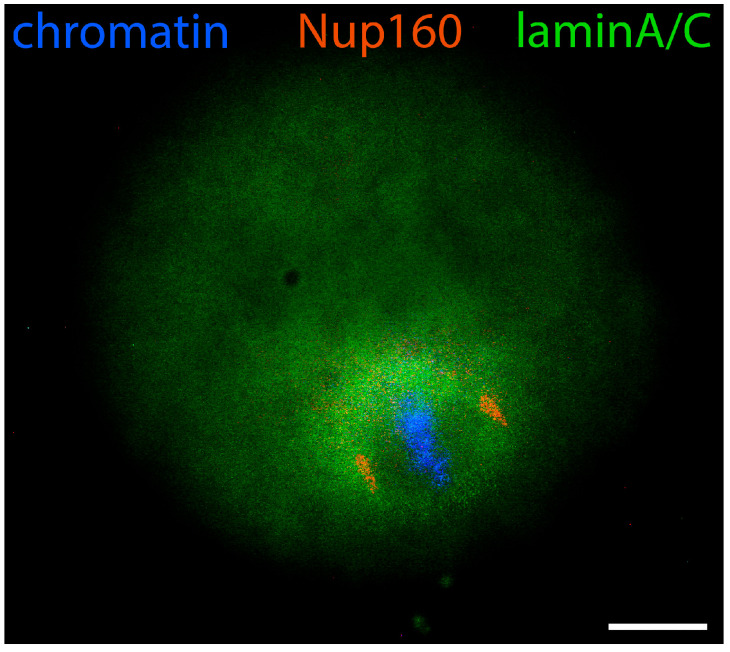
Mouse oocyte in metaphase I stained for lamin A/C, Nup160, and chromatin, epifluorescence. The cell was labeled as a part of the research described in [24]. Bar = 20 μm.

With the transition to metaphase I, and later to metaphase II, lamin B and Nup160 become associated with the meiotic spindle [24]. However, they do it in different ways: lamin B connects to the microtubules (Figure 12), similarly to its interaction with the mitotic spindle of somatic cells [39,40]. Nup160 localizes to the spindle poles while lamin A/C shows more diffuse localization in the ooplasm, but has a tendency to concentrate around the spindle (Figure 13).

**Figure 14:**
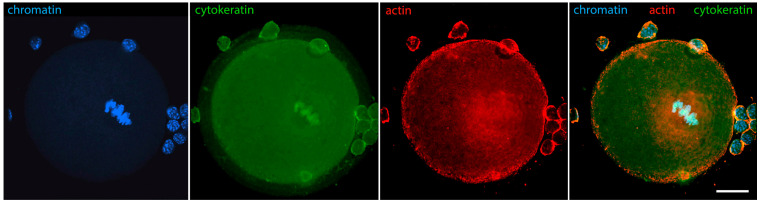
Mouse oocyte in metaphase I, reaction for cytokeratins, fibrillar actin, and chromatin, confocal microscopy. The cell was labeled as a part of the research described in [29]. Bar = 20 μm.

**Figure 15:**
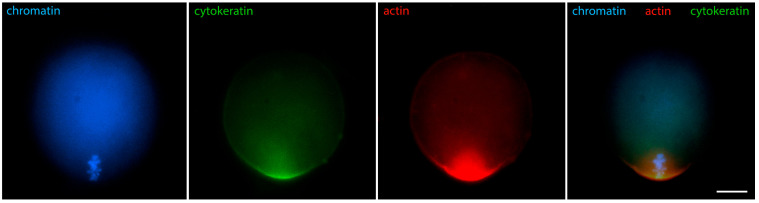
Mouse oocyte in metaphase I, reaction for fibrillar actin, cytokeratins, and chromatin, epifluorescence. The cell was labeled as a part of the research described in [29]. Bar = 20 μm.

In meiotic metaphase, IFs and microfilaments are both located around the spindle (Figure 14). There is, however, an important difference in the distribution of microfilament and IF components: cytokeratins and vimentin colocalize with the meiotic chromosomes, while actin is not found there. In addition, both types of fibers contribute to the spindle anchoring to the oolemma. In fact, the well-known actin cap also contains IFs, as can be seen in Figure 15. This distribution of IFs and microfilaments is valid for both metaphase I and II [29].

This way, in metaphase oocytes, the discussed key nuclear envelope and cytoskeletal proteins are found associated with the meiotic spindle (spindle fibers and/or spindle poles) and, in the case of cytoplasmic IF proteins, also with the condensed chromosomes themselves. This way, the proteins associated with the karyosphere during the late GV stage are later associated with the spindle during the MI and MII stages, a dynamic mimicking that of chromosomes themselves. In this respect, it is notable that oocyte chromosomes are important for meiotic spindle assembly due to the absence of centrosomes in the maturing oocytes [4]. We could hypothesize that the karyosphere serves as a hub organizing not only the meiotic chromosomes, but also the nuclear and cytoplasmic proteins that will later engage in the formation, positioning, or control of the meiotic spindle. It is known that the presence of condensed chromatin leads to an activity gradient of Ran-GTP that triggers microtubule nucleation in the vicinity, promoting spindle assembly [41]. Future studies may reveal analogous mechanisms underlying the reorganization of the other discussed proteins under the influence of the condensed karyosphere heterochromatin.

**Figure 16:**
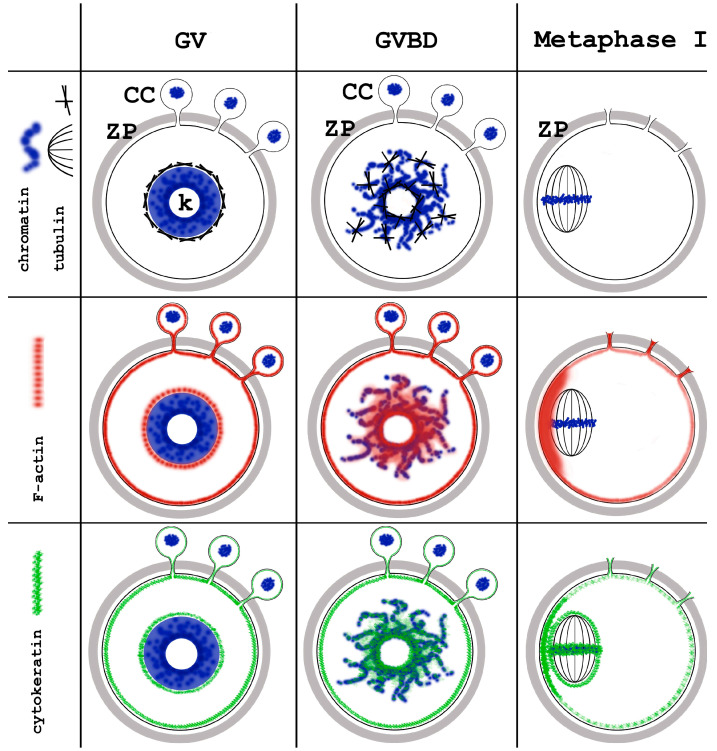
Summary of the dynamics of the main cytoskeletal proteins during meiotic chromatin rearrangement in mouse oocytes. GV, germinal vesicle; GVBD, germinal vesicle breakdown; ZP, zona pellucida; CC, cumulus cells; k, karyosphere.

**Figure 17:**
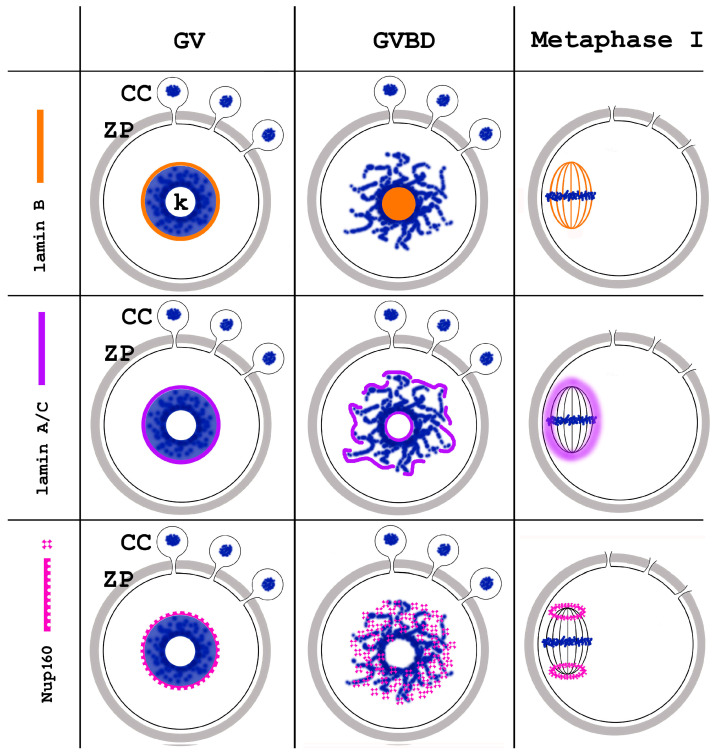
Summary of the dynamics of the selected nuclear envelope proteins during meiotic chromatin rearrangement in mouse oocytes. GV, germinal vesicle; GVBD, germinal vesicle breakdown; ZP, zona pellucida; CC, cumulus cells; k, karyosphere.

The changing intracellular distribution of the cytoskeletal proteins during mouse oocyte meiotic maturation is summarized in Figure 16, and that of nuclear envelope proteins in Figure 17.

## 8. Conclusions

Numerous studies have shown a correlation between the karyosphere status of GV mouse oocytes and the structural and functional state of their nuclear envelope, including its permeability. Through mechanisms that remain to be clarified, proteins from the nuclear periphery and the cytoplasm are recruited to the karyosphere. They include nuclear lamins, Nup160, actin, tubulin, and cytoplasmic intermediate filament components. These important structural proteins associate with the chromosomes and, after GVBD, colocalize with the meiotic spindle and may contribute to its formation and dynamics. The known correlation of the morphological presence of a karyosphere (SN status) of the oocyte and its developmental competence could reflect a role of the karyosphere in the organization, not only of the chromosomes, but also of the proteins that are important for successful meiotic maturation, fertilization, and early embryonic development.

## Data Availability

No new data were created or analyzed in this study.
